# Sports in Natural Forests: A Systematic Review of Environmental Impact and Compatibility for Readability

**DOI:** 10.3390/sports13080250

**Published:** 2025-07-29

**Authors:** Iulian Bratu, Lucian Dinca, Ionut Schiteanu, George Mocanu, Gabriel Murariu, Mirela Stanciu, Miglena Zhiyanski

**Affiliations:** 1 Department of Agricultural Sciences and Food Engineering, “Lucian Blaga” University of Sibiu, 7-9 Dr. Ion Ratiu Street, 550024 Sibiu, Romania; mirela.stanciu@ulbsibiu.ro; 2 National Institute for Research and Development in Forestry “Marin Dracea”, Eroilor 128, 077190 Voluntari, Romania; ionut.schiteanu@icas.ro; 3 Faculty of Physical Education and Sport, “Dunarea de Jos” University of Galati, 800008 Galati, Romania; george.mocanu@ugal.ro; 4 Department of Chemistry, Physics and Environment, Faculty of Sciences and Environmental, “Dunarea de Jos” University of Galati, 47, Domneasca Street, 800008 Galati, Romania; gabriel.murariu@ugal.ro; 5 Forest Research Institute—Bulgarian Academy of Sciences, 1753 “St. Kliment Ohridski” Blvd., 1756 Sofia, Bulgaria; miglena.zhiyanski@gmail.com

**Keywords:** forest ecosystems, sports, forest recreation, environmental impact, bibliometric analysis

## Abstract

The intersection of sports and natural forests and green spaces represents an emerging interdisciplinary field with implications for public health, environmental science, and sustainable land management and refers to the variety of cultural ecosystem services demanded by people from ecosystems. This manuscript presents a systematic bibliometric and thematic analysis of 148 publications for the period 1993–2024 identified through Web of Science and Scopus, aiming to evaluate the current state of research on sports activities conducted in natural forest environments. Findings indicated a marked increase in scientific interest of this topic over the past two decades, with key contributions from countries such as England, Germany, China, and the United States. Researchers most frequently examined sports such as hiking, trail running, mountain biking, and orienteering for their capacity to provide physiological and psychological benefits, reduce stress, and enhance mental well-being. The literature analysis highlights ecological concerns, particularly those associated with habitat disturbance, biodiversity loss, and conflicts between recreation and conservation. Six principal research themes were identified: sports in urban forests, sports tourism, hunting and fishing, recreational sports, health benefits, and environmental impacts. Keyword and co-authorship analyses revealed a multidisciplinary knowledge base with evolving thematic focuses. In conclusion, the need for integrated approaches that incorporate ecological impact assessment, stakeholder perspectives, and adaptive forest governance to ensure sustainable recreational use of natural forest ecosystems is underlined.

## 1. Introduction

A growing body of literature highlights the health benefits of engaging with natural environments [1,2]. Outdoor recreation in forests, parks, and other green spaces has been shown to promote physiological relaxation, enhance positive emotions, and restore attention [3,4,5]. These natural settings also serve as ideal spaces for physical activity, acting as a key mechanism linking green spaces to overall well-being [6,7,8].

A sustainable sports event is generally understood as one that aims to minimize environmental impact through measures such as waste reduction, energy efficiency, and sustainable transportation, while also promoting social goals like inclusivity, diversity, community engagement, and economic viability [9]. In line with this, the Active Forest Programme was launched as a collaboration between Sport England and Forestry England, with the goal of encouraging people to engage in enjoyable physical activities within England’s public forest estate [10].

Forest-based activities offer a dual benefit: exposure to a natural environment and the physical activity itself [11]. Kobayashi et al. [12] compared the effects of a 15-min walk in both forested and urban areas with passive viewing of these landscapes. The results indicated that walking in nature significantly reduced fatigue and confusion while increasing vigor. Similarly, Hansmann et al. [13] examined the restorative effects of various activities in green spaces and found that sports such as jogging and cycling were associated with increased levels of positive affect compared to moderate activities like walking.

Many sports that are increasing in popularity take place in natural settings, where participants not only engage in physical activity but also appreciate the intrinsic values of nature, wildlife, and cultural heritage [14]. Two such disciplines that have gained significant traction are hiking and mountain trail running. Hiking is widely accessible and requires minimal financial investment, making it an inclusive activity for people of all ages. In contrast, mountain trail running and trail races were initially designed for experienced runners with challenging courses, but their popularity has expanded in recent years [15].

The concept of forests as sports venues raises an interesting comparison with traditional stadiums. A forest meets several criteria associated with a sports venue, as defined by sports regulations. It is a public or private asset designed or utilized—either permanently or temporarily—for sporting activities. Many forests, especially those near urban areas, are equipped with facilities to accommodate outdoor sports. However, cities are more vulnerable to climate change, and integrating green infrastructure—such as accessible forested areas for recreational use—into long-term urban planning is essential to enhance urban resilience, reduce stress, and promote sustainable well-being [16,17,18,19]. While access to state-owned forests is typically free, private forests often offer paid recreational activities such as tree climbing and paintball. More broadly, natural landscapes, including mountains, can be considered outdoor stadiums, serving as both recreational spaces and tools for performance measurement [20].

The term “natural forests” refers to ecosystems that have regenerated naturally, consisting of indigenous or naturally immigrated tree species. These forests are among the most complex terrestrial ecosystems in terms of biodiversity, ecological functions, and structural stability [21]. Various definitions of natural forests exist, with the minimum level of naturalness typically defined as naturally regenerated native forests, regardless of the intensity of human use [22,23,24,25].

Bibliometric analysis is a statistical method used to assess the impact of research within a given field. The development of advanced bibliometric software has made it increasingly accessible for researchers to analyze trends, identify foundational knowledge, and predict future research directions. Compared to traditional literature reviews, bibliometric analysis enables scholars to rapidly gain insights into a field’s history and emerging topics, condensing what once took years of exploration into a more efficient process [26,27,28,29]. There are bibliometric studies in the field of sports [30,31,32,33], as well as in the field of forestry [34,35,36,37,38]. However, we did not identify any bibliometric study regarding sports activities in natural forests, which is why we wrote this review.

Despite this growing interest in both natural forest ecosystems and outdoor sports, there is a notable lack of comprehensive, interdisciplinary research that systematically examines their intersection. Current literature tends to focus either on the health benefits of nature exposure, the ecological impact of recreation, or sports participation trends in isolation. Few studies assess how sporting activities specifically conducted in natural forests influence ecological integrity or how forest characteristics affect the quality and sustainability of these sports experiences. Moreover, the compatibility between recreational sports and conservation goals in natural forest settings remains poorly understood, limiting the ability of policymakers and land managers to make evidence-based decisions.

To address this gap, the goal of our study was to conduct a systematic analysis of existing research on the relationship between sports activities and natural forests. This study aimed to identify emerging trends, key contributors, and thematic areas of research within this interdisciplinary field. By analyzing scientific publications, we sought to understand the compatibility of sports activities with forest conservation, the environmental impact of outdoor recreation, and the benefits of engaging in sports within natural forest ecosystems. Through this comprehensive review, we hope to contribute valuable insights for policymakers, researchers, and practitioners working at the intersection of sports, environmental sustainability, and forest management.

## 2. Materials and Methods

The bibliographic database for this study was compiled using the Web of Science Core Collections, accessed via the Web of Science (WOS) platform [39]. Clarivate, a global leader in transformative intelligence, provides enriched data, insights, analytics, workflow solutions, and expert services across academia and government, intellectual property, and life sciences and healthcare. Recognized as the most trusted and publisher-independent database, WOS is widely utilized in bibliometric research for its reliability and comprehensive coverage. Using WOS’s “Advanced Search” feature, searches were conducted with the keywords “sports and natural forests”, focusing on publications from 1 January 1993, to 31 December 2024. We also utilized the Scopus database of published articles. We excluded articles duplicates in both of the databases, articles without abstract, articles not written in English and articles not relevant for the topic and in final remained 148 articles. Data processing involved Web of Science Core tools, Microsoft Excel [40], and Geochart [41]. For mapping visualizations and cluster analysis, VOSviewer (version 1.6.20) was employed [42]. This bibliometric analysis aimed to identify emerging trends, key contributors, and insights regarding relevant articles, authors, and journals. The study examined ten primary aspects: publication types; Web of Science categories; publication years; countries; institutions; language; journals; publishers; authors; keywords.

A key component of the study was evaluating an article’s Total Link Strength, which, along with the Links attribute, serves as an indicator of influence. The Links attribute quantifies an article’s direct connections with others, while Total Link Strength measures the overall strength of these connections, factoring in co-authorship networks. In co-authorship analysis, the Links attribute represents the number of direct collaborations a researcher has, whereas Total Link Strength reflects the cumulative weight of these co-authorship ties within the academic community. This distinction is crucial for identifying research teams and understanding how collaborations shape scientific contributions.

**Figure 1:**
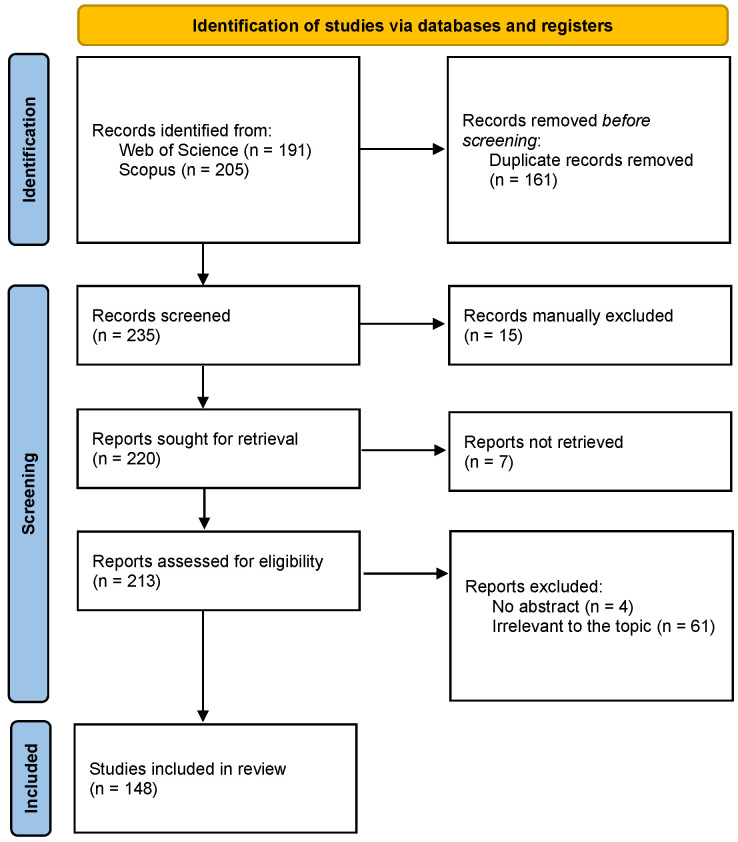
Selection process of the eligible reports based on the PRISMA 2020 flow diagram.

We followed the Preferred Reporting Items for Systematic Reviews and Meta-Analyses (PRISMA) guidelines [43]. The selection process of the papers included in this review is illustrated in Figure 1. Finally, 422 papers were included in this systematic review (Figure 1).

**Figure 2:**
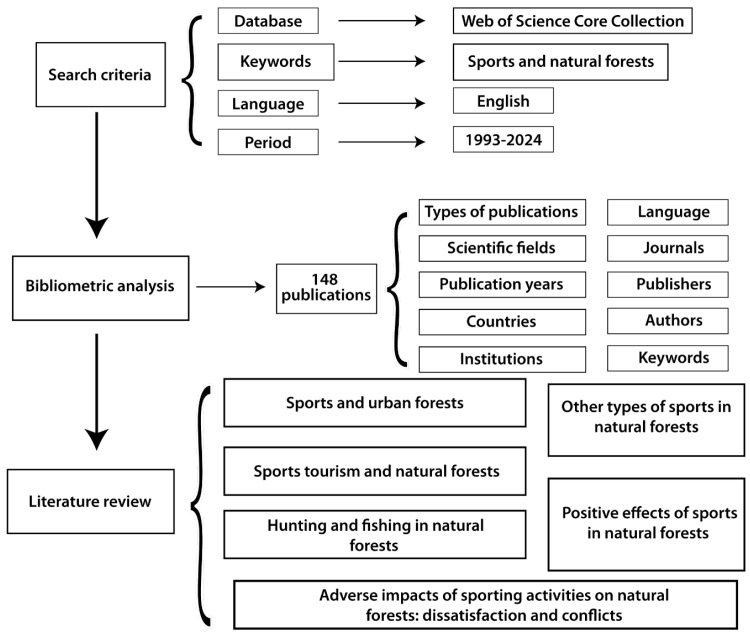
Schematic presentation of the workflow.

The second phase of the study involved a literature review of 148 articles, which were categorized into six main groups: sports and urban forests; sports tourism and natural forests; hunting and fishing in natural forests; other types of sports in natural forests; positive effects of sports in natural forests; adverse impacts of sporting activities on natural forests: dissatisfaction and conflicts. Figure 2 illustrates these categories and their interconnections.

## 3. Results

### 3.1. Bibliometric Analyses

**Figure 3:**
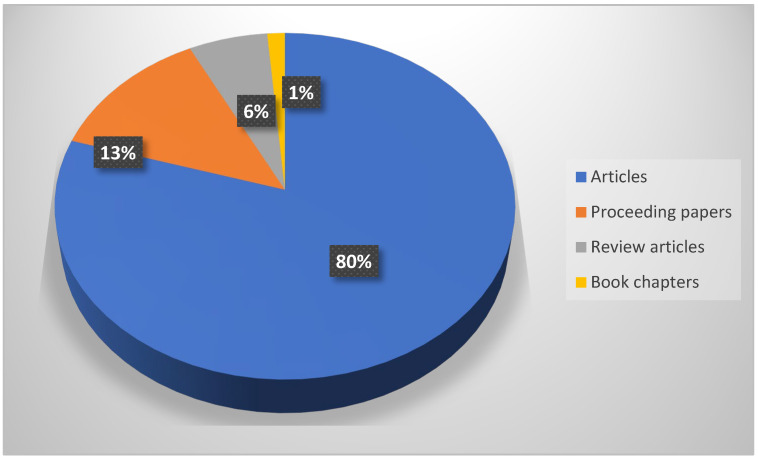
Distribution of the main types of publications used in the bibliometric analysis.

Up until 2024, a total of 148 publications have been published on the topic of sports and natural forests. These were distributed as follows: 118 were articles (80% of the total publications), 19 were proceeding papers (13%), 14 were review articles (6%), and 2 were book chapters (1%) (Figure 3).

**Figure 4:**
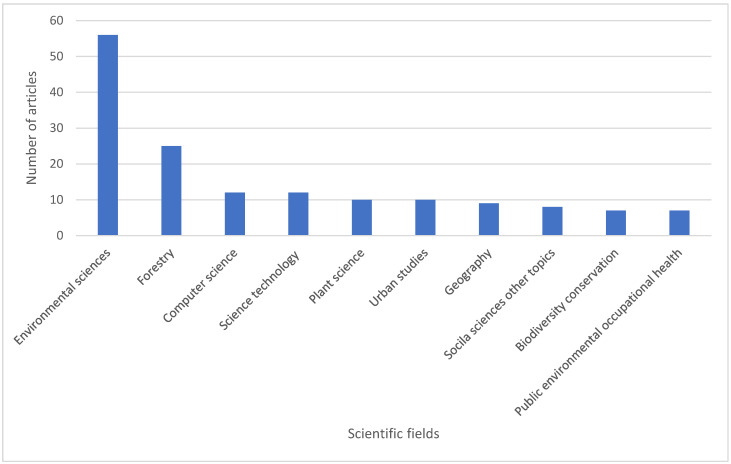
Distribution of the main research areas of publications used in the bibliometric analysis.

The total number of published articles can be grouped into several research areas, according to Web of Science classification. In our study, we identified 52 research areas, with the most representative being environmental sciences and ecology (56 articles), forestry (25 articles), computer science (12 articles), and science and technology—other topics (12 articles) (Figure 4).

**Figure 5:**
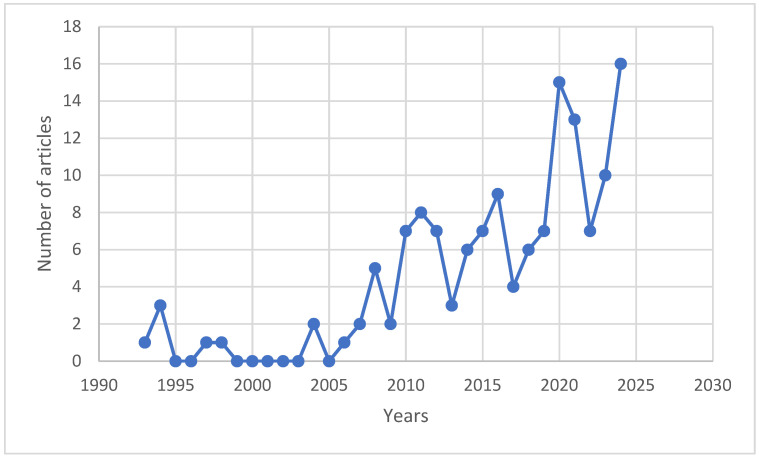
Distribution of articles concerning sports and natural forests by year.

**Figure 6:**
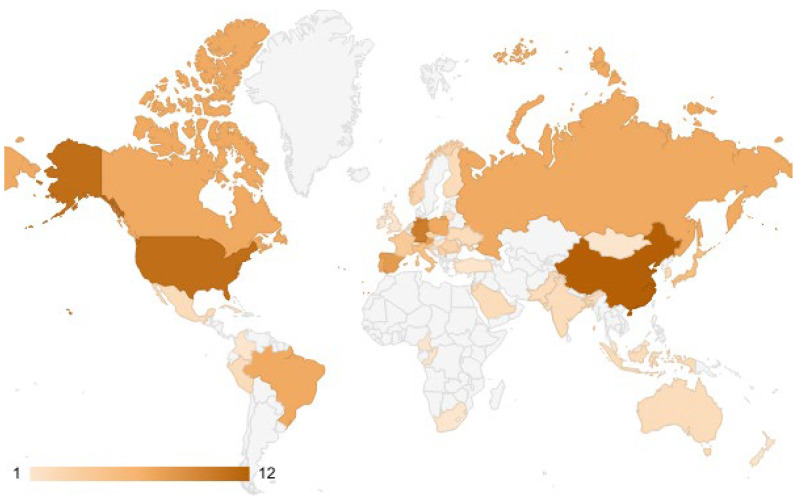
Countries with authors of articles on sports and natural forests.

Author contributions were relatively evenly distributed: only one author, Liz O’Brien, authored three articles, while nine other authors—Marco Isaia, Steeve Côté, Claudia Palestrini, Antonio Rolando, Christian Dussault, Andreas Matzarakis, Estela Inés Farías Torbidoni, Matteo Negro, and Xiaoe Zeng—each contributed to two articles. The number of articles published in articles concerning sports and natural forests has experienced significant growth over the past two decades (Figure 5). A total of 52 countries have been identified as the affiliations of authors publishing on this topic, spanning five continents (Figure 6).

Based on the number of published articles, the most representative countries were England (12 articles), China (12 articles), Germany, and the USA (each with 10 articles). Meanwhile, based on Total Link Strength, the ranking was England, Germany, and Portugal (Table 1). Total Link Strength is a measure of collaboration and research group effectiveness, determined by two key indicators: the Links attribute, which quantifies the number of direct connections an article shares with other publications; the Total Link Strength attribute, which assesses the overall intensity of these connections, particularly within co-authorship networks. For instance, in co-authorship analysis, the Links attribute reflects the number of direct co-author relationships, while Total Link Strength measures the overall strength of these collaborations within the academic landscape. This methodology helps identify research clusters and assess the role of scientific collaboration in advancing knowledge.

**Figure 7:**
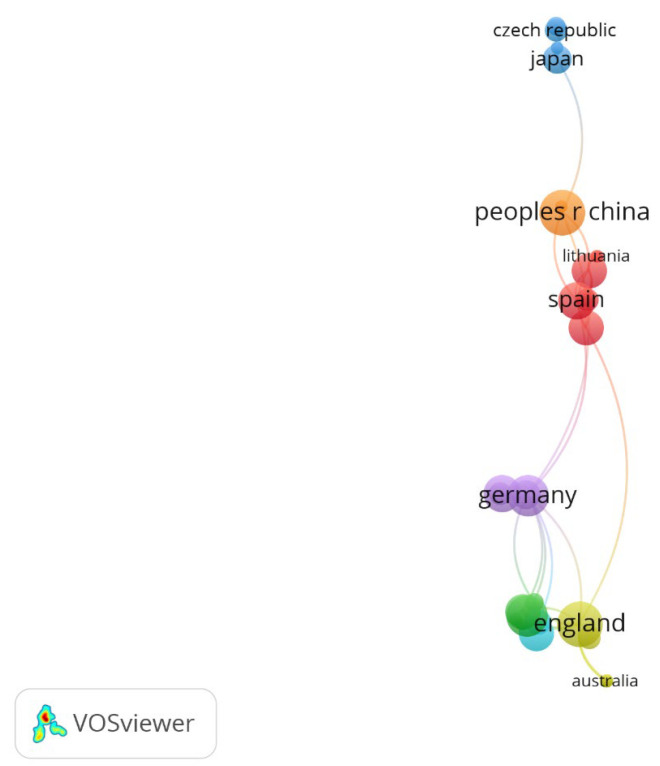
Clusters of countries with authors of articles on sports and natural forests.

The countries of origin of the authors who have published articles on sports and natural forests were grouped into seven clusters, four of which had a larger number of countries; the first cluster included Bosnia and Herzegovina, Lithuania, Portugal, Russia, Serbia, Spain, and Turkey; the second cluster included Brazil, Colombia, Indonesia, the Netherlands, and the USA; the third cluster included the Czech Republic, Finland, Japan, Malaysia, and Mongolia; and the fourth cluster included Australia, England, New Zealand, Norway, and Singapore (Figure 7).

The most representative institutions where authors published on sports and natural forests were affiliated include Laval University (with four published articles) and South Ural State University, the University of Fribourg, and the University of Ljubljana (each with three published articles) and were found in 113 journals, with the highest number of articles published in the following journals: urban forestry and urban greening (eight articles); sustainability and forests (each with six articles); forest ecology and management (four articles) (Table 2).

The majority of articles were published in English (129 articles). However, articles were identified in other languages: Polish, Russian, Spanish, and Turkish (each with three articles), French (two articles), and Croatian and Portuguese (each with one article).

A total of 61 publishers had published articles on this topic. The most representative were Elsevier (28 articles); MDPI (20 articles); Springer Nature (17 articles); Taylor & Francis (10 articles). The most frequently used keywords are *health*, *forest*, *biodiversity,* and *physical activity* (Table 3).

**Figure 8:**
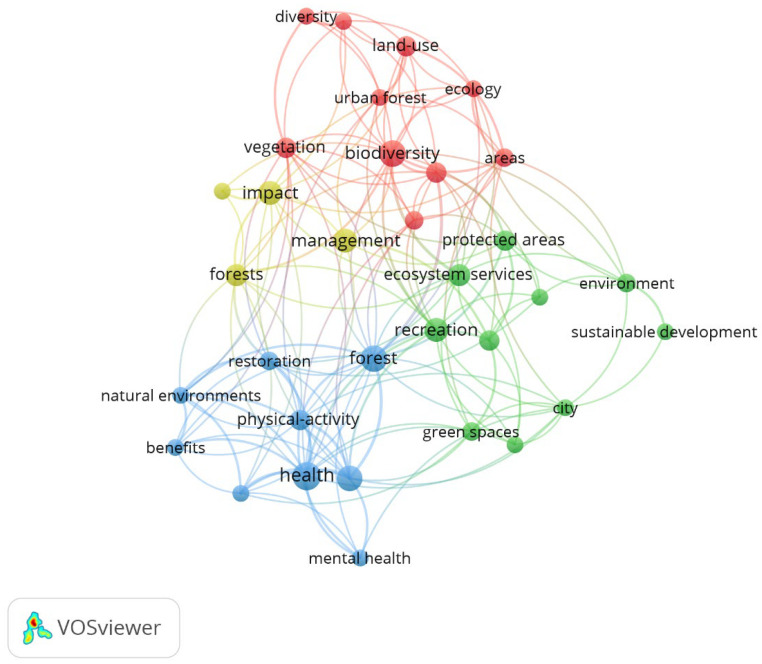
Authors’ keywords concerning sports and natural forests.

The keywords were grouped into four clusters, with the most important being Cluster 1, which had keywords such as areas, biodiversity, diversity, ecology, land-use, national park, soil, tourism, urban forest, and vegetation; Cluster 2, which had the keywords city, ecosystem services, environment, green spaces, preferences, protected areas, quality, recreation, sustainable development, and urban (Figure 8). These clusters reflected four interconnected thematic areas: ecological and land-use concerns (Cluster 1), urban and environmental planning (Cluster 2), human interaction and governance (Cluster 3), and health-related benefits and interventions (Cluster 4). This synthesis highlights the multidimensional nature of sports activities in natural forests, linking biodiversity and conservation with public health and urban sustainability.

**Figure 9:**
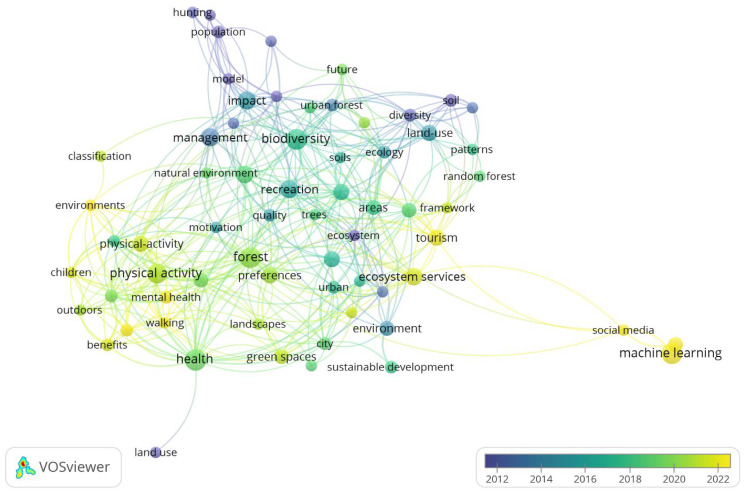
Distribution of keywords over time (periods) concerning sports and natural forests.

In the 2011–2014 period, commonly used keywords included population, model, diversity, soil, and ecosystem. In the 2015–2019 period, the most frequently used keywords were recreation, quality, health, areas, biodiversity, and patterns. In the 2020–2023 period, the most frequently used keywords were social media, machine learning, tourism, environments, and mental health (Figure 9).

### 3.2. Literature Review

#### 3.2.1. Sports and Urban Forests

Urban forests provide a variety of cultural ecosystem services, including recreation, social interactions, cultural heritage, and educational values. Human-made infrastructure, such as historic sites and sports facilities, as well as blue spaces, play a crucial role in enhancing these services. Many individuals experience cultural ecosystem services through active recreation, particularly sports [44].

##### The Relationship Between Green-Blue Spaces and Physical Exercise

Building on this, a growing body of research has explored how the composition and design of urban green and blue spaces influence patterns of physical activity. For instance, a study conducted in Singapore found that areas with higher proportions of forest and scrub cover encouraged more walking, while scrub-dominated areas were associated with reduced activity. Team sports were more frequently practiced in spaces with open-canopy managed vegetation, whereas individual exercises were more prevalent in areas combining open-canopy vegetation with adjacent blue spaces. Similarly, a positive correlation was found between physical activity levels and residential proximity (within 500 m) to a mix of managed treescape and forest cover [45].

##### Health Benefits of Exercising in Natural Outdoor Environments

These spatial factors also intersect with the health benefits of exercising in natural outdoor environments (NOEs), which include urban forests and blue spaces. The presence of nature, fresh air, and open spaces in NOEs can enhance the quality of physical exercise, making it more enjoyable and restorative. This, in turn, contributes to improved mental well-being [46,47]. Research supports the role of NOEs in facilitating mental restoration [48] and increasing motivation for physical activity [49,50]. Moreover, NOEs offer accessible venues for routine activities such as jogging or team sports, integrating exercise into daily life with ease [51]. Nevertheless, current research often either narrows its focus to specific NOEs like urban forests or uses broad, undifferentiated categories such as parks [52], suggesting a need for greater specificity in future studies.

##### Global Perspectives on Urban Forests and Sports

The significance of urban forests in supporting sports and recreational activities is evident across diverse global contexts. For example, the Belgrade Forest nature parks in Istanbul attract visitors due to their cleanliness, family-oriented environment, tranquility, and suitability for sporting activities [53]. Conversely, a study in an urban forest park in China found that most visitors engaged in passive recreation, with lower participation in active sports and learning-related activities [54]. Similarly, big data analysis from Seoul indicated that integrating “hard fascinations”—including sports, entertainment, and educational features—could enhance the restorative benefits of urban forests [55].

Further examples illustrate the multifunctionality of urban forests in different settings. Germia Park in Kosovo provides opportunities for sports, leisure, meditation, and biodiversity conservation, reflecting a balance between human use and environmental values [56]. In Australia, park renovations such as improved walking paths and play facilities led to increased user numbers and physical activity [57]. However, outcomes have not been universally positive: some studies found no significant changes in activity levels following similar improvements [58,59], and in some cases, even reported declines [58]. These varied findings underscore the complexity of designing urban forests that effectively promote both passive and active forms of engagement. A more nuanced understanding of user needs, environmental design, and cultural context is therefore essential in maximizing the recreational and health benefits of these spaces.

#### 3.2.2. Sports Tourism and Natural Forests

Sports tourism plays a vital role in the broader tourism industry, encompassing activities centered around sports and physical engagement [60]. It includes various forms of fitness, recreation, adventure, rehabilitation, and cultural sports exchanges, such as mountaineering, rock climbing, adventure sports, forest tourism, and participation in sporting events, including traditional national sports [61]. As the foundation of the sports tourism industry, sports tourism resources provide opportunities for sightseeing, leisure, adventure, fitness, rehabilitation, and competitive sports [62].

The growing emphasis on health and well-being has strengthened the connection between tourism, recreation, and sports within natural environments, particularly in forested areas. Research indicates that individuals under the age of 30 and those over 45 are the most active participants in sports like orienteering. Their primary motivations include a desire to immerse themselves in nature, relieve stress, breathe clean air, and appreciate scenic landscapes [63].

An example of integrating sports tourism with environmental conservation is the post-fire landscape rehabilitation initiative in the protected area of Montejunto, Portugal. One of its key objectives is to promote ecotourism and outdoor sports while preserving the area’s natural, historical, and cultural identity to ensure long-term sustainability [64].

To minimize the environmental impact of sports tourism, the advancement of ecologically responsible sports tourism—grounded in ecological principles—has become a strategic priority for ensuring its sustainable and healthy development. The expansion and pace of sports tourism are influenced not only by the appeal of natural and recreational resources but also by the broader economic and social development of the region. To be sustainable, the supply of sports tourism must be aligned with market demand, integrating sports and tourism in a synergistic, ecologically sound manner. This involves designing diverse and environmentally friendly sports activities that meet tourist needs while delivering ecological, economic, and social benefits [65].

Ecotourism, often associated with “slow tourism”, is increasingly recognized as a tourism model most aligned with sustainable development goals. This form of tourism emphasizes low-impact, environmentally conscious travel, particularly within protected natural areas, and promotes behavior that contrasts with mass tourism. Ecotourism in such areas fosters environmental education, awareness, and cultural appreciation. Recent research highlights the significance of natural, historical, and cultural resources in creating ecotourism products, supported by interpretive and educational technologies. The role of trained guides in facilitating meaningful, low-impact engagement with nature is also emphasized [66].

Global ecotourism research has grown steadily since the late 1980s, driven by increasing awareness of its potential to contribute to biodiversity conservation and the socioeconomic development of local communities, particularly in developing countries. This research tends to focus on national parks in Asia and Africa, which contain globally significant biodiversity hotspots. Recognizing these benefits, many nations are now integrating ecotourism—often in conjunction with recreational and sport-oriented activities—into their sustainable development strategies [67].

Further evidence suggests that effective ecotourism, especially when linked to sport events, requires attention to broader social and political factors beyond standard tourism metrics. Indicators such as education, literacy, local governance, and political agendas play a vital role in shaping the successful implementation of ecotourism practices associated with sport activities. These elements help distinguish authentic ecotourism initiatives from more commercialized or less sustainable models [68].

#### 3.2.3. Hunting and Fishing in Natural Forests

Hunting and fishing play a significant role in managing wildlife populations and supporting conservation efforts in natural forests. Below, we summarize key research findings on the impact of hunting and fishing on animal population control, ecosystem balance, and socioeconomic benefits.

##### Wildlife Population Control and Ecosystem Management

Sport hunting has been recognized as an effective tool for controlling cervid populations over large areas. Similar to natural predators, the efficiency of sport hunting is influenced by various environmental factors. A deeper understanding of these variables can aid in developing more effective wildlife management strategies [69].

Hunting activities can also help mitigate forest damage caused by herbivores. For example, in Bavaria, Germany, the implementation of a large-scale game management system led to a reduction in browsing damage, which facilitated unfenced forest regeneration. Areas where deer harvests increased in accordance with 2006 game management plans experienced a notable decline in browsing damage by 2009 [70]. Similarly, a large-scale study in Sweden demonstrated that reductions in moose populations, as indicated by harvest data, corresponded with decreased browsing damage in forest inventories [71].

In recent years, conservation strategies in boreal and temperate forests across Europe and North America have shifted focus. Rather than solely managing habitats to increase deer carrying capacity and enforcing restrictive hunting regulations, efforts now prioritize reducing deer impacts on ecosystems and promoting sustainable deer populations [72,73]. Decision-making in deer-forest management requires predictive models that account for multiple ecological and population parameters.

Overabundant cervid populations have led to severe negative effects on plant communities worldwide. Antlerless deer harvest by sport hunters has been proposed as a potential solution to overabundance. The philopatric behavior of female deer is expected to limit recolonization in heavily hunted zones, thus contributing to population control [74].

##### Hunting and Conservation Efforts

The role of sport hunting in conservation remains a contentious issue within environmental management discourse. On one hand, several case studies suggest that legal, well-regulated sport hunting can support conservation objectives while offering economic incentives to local communities. For instance, in Mexico, species such as white-tailed deer, mule deer, red brocket, and brown brocket are not only central to the subsistence diets of indigenous and rural populations but also serve as key resources for sport and trophy hunting. The most robust populations of these species are often found in protected areas, which function as genetic reservoirs and source populations for surrounding regions. These areas support a dual-use model wherein local communities can both harvest animals for subsistence and benefit from tourism and biodiversity conservation programs [75].

Similarly, in North America, black bear (*Ursus americanus*) population management uses controlled sport harvest as a cost-effective strategy to maintain population viability while reducing the frequency of human–bear conflicts. Strategic harvest management is employed to balance population control with conservation objectives [76].

In the Republic of Cameroon, the Lobéké National Park has established community hunting zones where subsistence and sport hunting coexist. These zones provide local protein sources and also generate income through sport hunting leases, which fund community development projects and foster support for conservation efforts [77].

However, the controversy lies in the ethical, ecological, and enforcement challenges surrounding sport hunting. Critics argue that the commodification of wildlife through trophy hunting can undermine intrinsic conservation values, especially when regulation is weak or governance is corrupt. Illegal sport hunting remains a critical threat to prey species, particularly when it targets reproductively significant individuals or overlaps with protected zones. Therefore, while evidence supports the role of sport hunting as a potential conservation tool, its success depends heavily on governance quality, transparent benefit-sharing, scientific monitoring, and rigorous law enforcement [78]. As such, sport hunting should not be universally promoted as a conservation strategy without careful consideration of local ecological, socioeconomic, and ethical contexts.

##### Economic Contributions of Fishing in Natural Forests

The economic benefits of recreational, commercial, and subsistence fishing on lands managed by the U.S. Forest Service are considerable and are expected to grow over time. Recreational fishing on national forests and grasslands generates over US$2.2 billion annually. These revenues—derived from fishing equipment, boats, travel, guiding services, fuel, and licenses—help fund critical fisheries habitat management and conservation efforts carried out by federal and state agencies [79].

In summary, hunting and fishing in natural forests serve as essential tools for wildlife population management, conservation, and economic sustainability. Properly regulated activities can mitigate ecological damage, support biodiversity, and provide significant socioeconomic benefits to local communities and broader conservation initiatives.

#### 3.2.4. Other Types of Sports in Natural Forests

An enumeration of various sports practiced in natural forests, as found in previously published articles, is presented in Table 4. A total of 17 different sports were registered in the published artcles with authors especially from Europe, but also from Asia and from Americs.

Following the data presented in Table 4, it is evident that natural forests support a remarkably diverse range of sports activities, spanning from low-impact, non-mechanized activities (e.g., hiking, jogging, orienteering) to more intensive or infrastructure-dependent practices (e.g., motor sports, kayaking, mountain biking). Hiking and running, frequently cited across multiple countries, appear to be among the most common and accessible activities in forested areas, reflecting a global preference for endurance-based recreation. Meanwhile, specialized sports such as climbing, horse riding, and trail racing are typically reported in regions with suitable terrain and established tourism infrastructure. Notably, many articles group several sports under broad categories—such as “ball sports” or “water sports”—indicating a tendency to frame forest use in terms of recreational diversity rather than singular sport types. This heterogeneity suggests a growing multifunctionality of forest spaces, where passive, active, individual, and group sports coexist, often with overlapping environmental implications.

#### 3.2.5. Positive Effects of Sports in Natural Forests

Research indicates that engaging in physical activities in green spaces enhances enjoyment, fosters revitalization, and positively impacts self-reported mental well-being [91]. Studies suggest that a significant proportion of individuals (89–92%) associate physical activity in natural forests with benefits such as improved physical well-being, a sense of fun and enjoyment, enhanced mental health, and a feeling of freedom [10].

Regular exercise in urban forest parks has been found to support endocrine and neurochemical regulation, effectively reducing urban stress and anxiety (MEA). Furthermore, participating in a combination of physical activities—including hiking, sports, and relaxation—yields a higher level of positive affect compared to engaging solely in relaxing activities [92].

Additionally, government initiatives aimed at increasing youth participation in outdoor sports have demonstrated the value of non-traditional activities in natural settings. A study on a mountain biking program in a forest in southeast England found that such initiatives not only encourage physical activity but also provide young people with opportunities to engage with nature in a way that aligns with their identities and active lifestyles [86]. In this context, natural forests function as “outdoor stadiums”—multifunctional spaces that not only host diverse physical activities but also support mental restoration and social connection, reinforcing their role as integral elements of sustainable urban and peri-urban environments.

#### 3.2.6. Adverse Impacts of Sporting Activities on Natural Forests: Dissatisfaction and Conflicts

In Portugal, natural parks are significantly impacted by various human activities, including camping, motor sports, the spread of invasive species, forest fires, and, along the coast, urbanization and tourism development. Motor sports, in particular, have been identified as a major factor contributing to environmental degradation [87].

In Turkey, Ilgaz Mountain National Park has seen a steady increase in visitors due to its popularity for winter tourism. However, this rising number of tourists has heightened the risk of environmental deterioration due to the park’s fragile natural structure [93]. Research in a natural forest area in Turkey revealed that nitrification and nitrogen mineralization levels were higher in areas where natural recovery had begun within ski runs. However, in severely damaged ski run areas, water-holding capacity, organic carbon, and total nitrogen content decreased, leading to a decline in mineralization and nitrification rates [94].

A study of slope instability in a mountain forest landscape focused on a Winter Olympic Games 2014 venue that rapidly expanded from an undeveloped area into a major tourist resort. The results indicated that this rapid development exacerbated slope hazards, leading to an increase in instability processes [95]. Similarly, in the Alps, human activity has significantly disturbed the environment since the early 20th century. The construction of buildings, roads, and direct damage caused by skiers and machinery have severely impacted fragile ecosystems. Ski pistes, in particular, have been found to affect all ecosystem components, including soil, plant, and animal communities. The use of snow-grooming vehicles further exacerbates soil and vegetation degradation [96,97].

In Beijing, China, dissatisfaction among residents regarding park management has been noted, with 17.11% of reviews highlighting concerns about poor guidance, inadequate security services, and substandard service attitudes. Access and parking difficulties during peak times for sports and cultural activities were particularly problematic. These findings emphasize the need for improved ecological environments and better-managed sports facilities [84].

In Spain, the Mosquera Valley in Sierra de Espadán Natural Park has experienced an increase in mountain races, raising concerns over environmental degradation. Park managers have thus far limited their response to recommending good practices and monitoring potential impacts such as erosion and pollution. Some measures, such as rerouting racecourses to minimize environmental damage, have been suggested to mitigate the conflict between recreational use and nature conservation [15].

Regarding orienteering in natural forests, key challenges include a lack of marked trails, long distances from designated paths, and time constraints. Participants are aware of the environmental impact of their activities, such as plant damage, undergrowth trampling, informal trail creation, wildlife disturbance, and littering. This awareness reflects a growing concern among orienteering practitioners about the negative effects of sports competitions on forest ecosystems [63].

Mountain biking management in natural areas faces challenges, particularly conflicts with other user groups such as hikers. Research from Slovenia suggests that effective management should include legislative measures to regulate trail access, the creation of single-use trails for different biking styles, and educational initiatives to promote responsible riding and environmental stewardship [98].

## 4. Discussion

This review aimed to address a notable gap in the scientific literature: the absence of a comprehensive, interdisciplinary analysis of the interactions between sports activities and natural forests. While prior studies have separately examined the health benefits of outdoor recreation or the ecological impacts of forest use, few have systematically assessed the compatibility between sporting activities and conservation goals in natural forest environments. The rationale for this review stems from the growing popularity of nature-based sports, increasing environmental concerns, and the need for evidence-based guidance for sustainable forest recreation. The primary purpose of our review was to provide a bibliometric and thematic synthesis of existing research, identify major trends and challenges, and propose directions for future study and policy.

### 4.1. Existing Literature on Sports and Natural Forests

The number of publications on sports and natural forests, e.g., 148, seems impressive and impacted the authors of this paper. Of course, the majority of publications were articles (80%), but the large number of proceeding papers (13%) is surprising, likely due to the numerous conferences that have addressed this subject. Among the nine review articles found in the database, those (weak) related to our topic were Hiking: A low-cost, accessible intervention to promote health benefits [99]; Progress on relationship between natural environment and mental health in China [100]; Is hunting an effective tool to control overabundant deer? A test using an experimental approach [74].

Additionally, the identification of 52 research areas is a high number, in consensus with the multidisciplinary nature of the analyzed topic, like in other review articles [101,102,103]. Interestingly, sports do not appear among the top research fields—instead, environmental topics dominate, which is justified given our search term (natural forests).

Regarding the countries from which the authors originate, we observed that England, Germany, China, and the USA—countries with a strong interest in sports—ranked highest. Meanwhile, African countries are poorly represented. The distribution of published articles per author or journal is relatively uniform, with very few authors or journals having a significantly higher number of publications on this topic. An interesting aspect is the evolution of keywords over time. In the initial stages, the keywords were general terms related to the topic. However, in recent years, there has been a growing prevalence of terms linked to modern environmental and human health concerns (e.g., social media, machine learning, environments, and mental health).

### 4.2. Different Types of Sports Practiced in Natural Forests

Nature sports refer to physical activities in which natural features—such as terrain, water, or weather—assume a central role, similar to the function of human competitors or partners in traditional sports [104,105]. These activities offer novel challenges and perspectives, diverging from conventional sports by engaging participants in alternative ways of seeing, doing, and understanding sport [106]. Originating primarily in North America during the 1960s and 1970s, nature sports have since evolved in response to emerging social values and demands for active, meaningful leisure, significantly reshaping the landscape of modern sport [107,108,109]. However, we only studied a small component of nature (natural forests) where sports are practiced. Nevertheless, the 17 sports identified were significant, especially considering that in some cases, we referred to categories of sports, such as ball sports, bat and racket sports, motor sports, and water sports. According to studies carried out in Spain by Múgica [110], Farias [111] and Muñoz [112], more than 60% of the visitors to protected natural areas do some kind of physical or sporting activity during their visit, and hiking is the most popular pastime (45%).

Hunting and fishing are also considered sports [113], even though that “It is argued here that enthusiasm for these sports is historically complex and relates to deeply embedded discourses on anti-modernism, neo-Darwinism, ecologism, and masculinity”. Far from being the preserve of traditional, rural groups in society, the new proponents of hunting and angling are drawn from sections of the urban middle class for whom such discourses have particular appeal [114]. It is natural for these sports to take place in nature, as hunting is strongly linked to forests and fishing to mountain streams in some cases.

Hunting wildlife has been common practice in human societies throughout history [115]. People still hunt wild animals for food and for income through trade in meat or byproducts [116], and predators are hunted to protect people, crops, and livestock [117]. Worldwide, many people hunt for recreation (i.e., sport hunting) [118], which includes trophy collection (e.g., antlers) [119]. Although sport hunting can have negative impacts on prey animal behavior, fitness, and population dynamics and thus lead to a decline in species richness and abundance [120], strictly controlled and regulated sport hunting can contribute to biodiversity conservation through generation of income for local communities, support of environmental agencies, and control of invasive species [118].

### 4.3. Sports and Urban Forests

Urban forests serve as integral spaces for recreation, fostering both passive and active engagement. The findings align with existing literature that highlights how urban green and blue spaces provide cultural ecosystem services (CES), particularly through sports and recreational activities [121,122,123]. The presence of human-made infrastructure, such as sports facilities and historic sites, further enhances these services by attracting individuals seeking opportunities for physical exercise. However, the extent to which these spaces promote active recreation varies significantly based on their design, accessibility, and composition.

This study’s results support the notion that the composition of urban green and blue spaces influences exercise choices [124,125,126]. As other authors have also observed, the variation in sports engagement across different urban forests emphasizes the influence of cultural, environmental, and infrastructural factors [76,127].

Infrastructure improvements in urban parks have demonstrated mixed results regarding physical activity levels. While some studies report increased engagement following enhancements [57,128], others indicate no significant change or even declines [59,129,130]. This highlights the complexity of designing interventions that effectively encourage long-term participation in physical activities. Factors such as accessibility, community involvement, and demographic differences likely contribute to these varied outcomes, necessitating further research to determine the most effective strategies for urban park development.

Our findings underscore the importance of intentional urban forest design to promote physical activity. Planners should integrate diverse vegetation structures, sports infrastructure, and interactive elements that cater to different recreational needs. Additionally, maintaining a balance between passive and active spaces is crucial to ensure inclusivity and accessibility. Further research is needed to explore the sociodemographic factors influencing engagement levels and to assess long-term trends in urban forest utilization for sports and exercise.

### 4.4. Positive and Negative Effects of Sports Practiced in Natural Forests

The positive effects of sports are undeniable. Moreover, sports performed in nature have beneficial effects on human health. Along with international efforts to utilize forests as preventive medicine, clinical trials on forest-based interventions have sharply increased. The effects of forest-based interventions have been reported in various health domains such as cardiovascular function [131,132,133], immune system [131,134,135], endocrine system [136] and mental health [137,138]. Others have focused on one specific health effect, such as blood pressure [139,140], diabetes [141], stress recovery [142], and depression [143].

Studies that highlight the broad range of benefits that can be gained from accessing forests [144] and other natural habitats [140,145]. The perceived mental well-being gained from being physically active also came out strongly in the evidence, similar to other studies [146,147,148]. The organized and led activities provided an opportunity to socialize with others, which has been found to be a benefit of forest and greenspace activity [149,150]. There is growing interest in the use of nature for physical activity and mental well-being and interventions in nature are of increasing interest to the environment, sport, and health sectors [151,152,153].

Sports generally have fewer negative effects on the environment. However, certain aspects associated with different sports may have notable environmental impacts. In particular, activities linked to sport infrastructure and maintenance can cause environmental degradation. For example, in the case of skiing, the establishment of ski-pistes affects every ecosystem component along a broad altitudinal range, encompassing both the montane and alpine belts (forest and treeless zones, respectively). Pastures or forest tracts are often abruptly clear-cut, and heavy machinery such as bulldozers and power shovels are used to remove soil and create suitable slopes for skiers. This process, commonly known as machine grading, is followed by artificial seeding to control soil erosion. These interventions significantly alter soil properties [154]. Machine-graded pistes typically exhibit lower vegetation cover, reduced species diversity, and a decline in the abundance of early flowering species [155]. Vegetation is further damaged by skiing activities and by the use of snow-grooming vehicles during ski-piste preparation [156]. The application of artificial snow prolongs snow cover, which in turn delays snowmelt and soil warming at the end of the season [157], potentially delaying vegetative re-sprouting [156]. Artificial snow can also introduce pollutants to the soil, including additives used to facilitate rapid and persistent freezing [156]. Additionally, during summer months, further environmental damage occurs as shrubs are cut and machine grading is performed to level rough or bumpy surfaces—an activity known as ground leveling—which continues to impact vegetation and soil [158,159].

### 4.5. Limitations and Future Research

Despite providing a comprehensive analysis of the relationship between sports activities and natural forests, our study has several limitations that should be acknowledged. Lack of in-depth environmental impact assessment: while our study categorizes research on the environmental effects of sports in natural forests, it did not conduct an ecological analysis or provide empirical data on specific impacts such as soil erosion, biodiversity loss, or habitat fragmentation. Further field-based research is needed to quantify these effects systematically. Evolving nature of outdoor sports: our study did not fully capture the rapid evolution of outdoor sports, including emerging disciplines such as e-biking, drone-assisted sports, and adventure racing. Many new activities are yet to be extensively studied in terms of their impact on forest ecosystems and recreational planning; Limited consideration of stakeholder perspectives: although the literature includes studies on public perceptions, the perspectives of key stakeholders—such as local communities, forest managers, conservationists, and policymakers—are underrepresented in bibliometric analyses. Future studies should integrate qualitative research methods, such as interviews and surveys, to gain deeper insights into these perspectives.

To address these limitations, future research should investigate new and emerging outdoor sports to assess their sustainability and compatibility with conservation efforts; incorporate stakeholder perspectives through qualitative research to understand the socioeconomic and cultural dimensions of forest-based recreation; encourage long-term monitoring of forest health and biodiversity in relation to recreational activities; and develop interdisciplinary studies that bridge the gap between sports science, environmental management, and policy-making. By addressing these gaps, future research can contribute to a more sustainable approach to integrating sports activities into natural forest ecosystems while ensuring their conservation and long-term viability.

## 5. Conclusions

A thorough bibliometric and literature-based analysis of the interdisciplinary field exploring the relationship between sports activities and natural forests demonstrates a growing academic interest. This trend is evidenced by the rising number of publications across various disciplines such as environmental sciences, public health, forestry, tourism, and urban planning. The analysis showed that diversity of sports practiced in natural forest environments ranges from hiking and mountain biking to hunting, fishing, and orienteering, but also that such diversity is linked to benefits and challenges associated with these activities. Forest-based sports are widely recognized for their contributions to physical fitness, psychological restoration, and social well-being. Numerous studies confirm that engagement in physical activity within forest environments enhances mental health outcomes, such as reduced stress, improved mood, and increased motivation to exercise. These benefits are amplified in comparison to similar activities in urban or indoor settings. Moreover, forests provide an inclusive and accessible facilities for recreation, offering opportunities for individuals of various age groups and socioeconomic backgrounds. Also, natural forests serve as important ecological assets, supporting biodiversity and delivering critical ecosystem services. The literature emphasizes that, without adequate planning and management, recreational use, particularly in the form of competitive or high-impact sports, can lead to negative environmental consequences at some extend. These effects include soil erosion, habitat fragmentation, vegetation damage, wildlife disturbance, and pollution. Motorized sports and winter tourism developments, for example, have been associated with long-term ecological degradation in fragile forest ecosystems.

The findings of this study focused on an urgent need for development of integrated, evidence-based strategies that balance recreational use with environmental conservation efforts. Interdisciplinary research is therefore essential to better understanding the complex interactions between sport, health, and forest ecosystems conditions. Furthermore, engagement of forest managers, local communities, recreational users, and policymakers is critical to developing context-specific and socially acceptable solutions.

From a methodological point of view, our study results illustrate the value of bibliometric tools in identifying research trends, leading authors and institutions, and emerging thematic areas. Our study also reveals some limitations in current research, including a lack of empirical studies assessing long-term environmental impacts, limited data on emerging outdoor sports, and insufficient incorporation of qualitative insights from affected communities.

The sustainable integration of sports into natural forests and green spaces requires a holistic and adaptive management approach that recognizes both the health-promoting potential of outdoor physical activity and the ecological sensitivities and vulnerability of forest ecosystems. Future research should prioritize long-term impact assessments, cross-sectoral collaboration, and the development of practical guidelines to harmonize human recreation with forest conservation goals.

## Figures and Tables

**Table 1:** The most representative countries where articles on sports and natural forests have been published.

Cur. no.	Country	Documents	Citations	Total Link Strength
1	England	12	232	11
2	Germany	10	307	10
3	Portugal	7	22	7
4	Scotland	4	59	7
5	Brazil	7	27	6
6	Spain	8	61	6
7	USA	10	164	6
8	Indonesia	2	11	5
9	China	12	63	5
10	Russia	7	19	5
11	Japan	5	89	4
12	Serbia	3	14	4
13	Austria	8	168	3
14	Canada	7	197	1
15	Italy	5	168	1
16	Poland	7	23	0

**Table 2:** The most representative journals where articles on sports and natural forests have been published.

Cur. no.	Journal	Documents	Citations	Total Link Strength
1	Urban forestry and urban greening	8	308	4
2	International journal of environmental research and public health	3	18	1
3	Sylwan	2	1	1
4	American journal of health promotion	1	2	1
5	International journal of advanced computer science and applications	1	31	1
6	Sustainability	6	27	0
7	Forests	6	5	0
8	Forest ecology and management	4	175	0
9	Biodiversity and conservation	3	92	0
10	Agroforestry systems	1	75	0
11	American journal of lifestyle medicine	1	58	0
12	Applied sciences	1	11	0
13	Ecosystem services	1	30	0

**Table 3:** The most commonly used keywords in published articles on sports and natural forests.

Cur. no.	Keyword	Occurences	Total Link Strength
1	forest	10	27
2	health	11	26
3	physical activity	9	24
4	biodiversity	10	23
5	natural environments	4	16
6	tourism	6	15
7	ecosystem services	7	14
8	restoration	5	14
9	areas	5	13
10	green space	4	13
11	recreation	8	13
12	vegetation	6	13
13	impact	8	12
14	preferences	6	12
15	land-use	6	11

**Table 4:** Articles citing different types of sports practiced in natural forests.

Crt. no.	Sports	Country	Authors
1	Ball sports	Switzerland	Hansmann et al., 2007 [80]
2	Bat and racket sports (e.g., table tennis, badminton)	England	O’Brien and Forster, 2020 [10]
3	Cycling	England	O’Brien and Forster, 2020 [10]
4	Climbing	Austria	Pröbstl-Haider et al., 2021 [81]
5	Cross-country races	Poland	Konieczny et al., 2020 [82]
6	Fitness (family fitness, boot camp, endurance)	USA; England; Austria	Cohen et al., 2012; O’Brien and Forster, 2020; Jungwirth et al., 2021 [10,58,83]
7	Hiking	France; China; England; Spain; Israel	Lepille, 2024; Zhang et al., 2024; O’Brien and Forster, 2020; Vidal-González and Vidal-Matzanke, 2020; Koniak et al., 2011 [10,15,20,84,85]
8	Horse riding	Poland	Konieczny et al., 2020 [82]
9	Jogging	Switzerland; Germany	Hansmann et al., 2007; Baumeister et al., 2020 [44,80]
10	Kayaking	China	Zhang et al., 2024 [84]
11	Mountain biking	France; Switzerland; Germany; England;	Lepille, 2024; Hansmann et al., 2007; Baumeister et al., 2020; King ang Church, 2015 [20,44,80,86]
12	Mountain trail running races	Spain	Vidal-González and Vidal-Matzanke, 2020 [15]
13	Motor sports	Portugal	Queirós, 2012 [87]
14	Orienteering	England; Poland; Australia	O’Brien and Forster, 2020; Mazurek-Kusiak and Soroka, 2021; Omodei and McLennan, 1994 [10,63,88]
15	Running	China; England	Zhang et al., 2024 [84]
16	Skying	Turkey	Aydınözü et al., 2011 [89]
17	Water sports (swimming, canoeing, fishing, boat ride)	Brazil	Nabout et al., 2022 [90]
